# Automated Mobile System for Accurate Outdoor Tree Crop Enumeration Using an Uncalibrated Camera

**DOI:** 10.3390/s150818427

**Published:** 2015-07-28

**Authors:** Thuy Tuong Nguyen, David C. Slaughter, Bradley D. Hanson, Andrew Barber, Amy Freitas, Daniel Robles, Erin Whelan

**Affiliations:** 1 Department of Computer Science, University of California, Davis, CA 95616, USA; 2 Department of Biological and Agricultural Engineering, University of California, Davis, CA 95616, USA; E-Mails: abarber@ucdavis.edu (A.B.); asfreitas@ucdavis.edu (A.F.); drrobles@ucdavis.edu (D.R.); emwhelan@ucdavis.edu (E.W.); 3 Department of Plant Sciences, University of California, Davis, CA 95616, USA; E-Mail: bhanson@ucdavis.edu

**Keywords:** tree crop enumeration, plant counting, uncalibrated camera, perspective transform, projection histogram, homography transform

## Abstract

This paper demonstrates an automated computer vision system for outdoor tree crop enumeration in a seedling nursery. The complete system incorporates both hardware components (including an embedded microcontroller, an odometry encoder, and an uncalibrated digital color camera) and software algorithms (including microcontroller algorithms and the proposed algorithm for tree crop enumeration) required to obtain robust performance in a natural outdoor environment. The enumeration system uses a three-step image analysis process based upon: (1) an orthographic plant projection method integrating a perspective transform with automatic parameter estimation; (2) a plant counting method based on projection histograms; and (3) a double-counting avoidance method based on a homography transform. Experimental results demonstrate the ability to count large numbers of plants automatically with no human effort. Results show that, for tree seedlings having a height up to 40 cm and a within-row tree spacing of approximately 10 cm, the algorithms successfully estimated the number of plants with an average accuracy of 95.2% for trees within a single image and 98% for counting of the whole plant population in a large sequence of images.

## 1. Introduction

To help cope with the rapid increase in the human population and future demands on worldwide food security, automation in agriculture is necessary. For example, there is a need to develop automatic systems for plant enumeration in fruit and nut tree seedling crops to save human resources and improve yield estimation. Most sensors used in agriculture have limited resolution, and cannot acquire the full scope of available plant and soil information. Advanced sensors, like cameras, that can characterize spatial and color information of natural objects play a crucial role in the future development of agricultural automation [1,2,3].

In the fruit and nut tree nursery industry, accurate counts of tree seedlings are very important in their production management and commerce [4,5]. Disease resistant tree rootstocks are planted from seeds in an outdoor nursery and they are later grafted to have fruit, which is a different cultivar from the disease resistant cultivar of the root system (*i.e.*, to combine the best features of two cultivars). Variability in germination rate and consumption by birds create uncertainty in the number of marketable seedlings available for sale. Traditionally, human workers must manually count the seedlings each spring after they have grown large enough to be safe from the birds and the final crop stand is stable. This method is slow, tedious, and costly for workers to perform. Additionally, while this method can be accurate when carefully conducted, in practice, human error and bias are still present and can lower the accuracy of the final count, particularly when workers get fatigued or distracted.

Recently, methods for plant population and spacing measurement using machine vision have been introduced for different kinds of plants. A daylight sensing system is presented in [4] to measure early growth stage corn populations. The algorithms used in the system include steps for image sequencing to merge information between consecutive video frames, vegetation segmentation using a truncated ellipsoidal decision surface and a Bayesian classifier, and plant counting based on the total number of plant pixels and their median positions. The image sequencing step in this study does not consider the case of a camera perspective change, however. In [5], algorithms for automatically measuring corn plant spacing at early growth stages are introduced. Plant morphological features, plant color, and the crop row centerline are among multiple sources of information utilized by the algorithms for corn plant detection and localization. This work points out that the estimated interplant spacing errors are due to crop damage and sampling platform vibration, which caused mosaicking errors. A machine vision-based corn plant spacing and population measurement system is presented in [6]. Algorithm steps in this paper include image sequencing using SIFT (Scale Invariant Feature Transform) feature matching, vegetation segmentation based on color channels, corn plant center detection using a skeletonizing algorithm, and calculation of corn spacing and plant count. This algorithm yields satisfactory results with images captured from the top view. In [7], we had proposed a mobile platform that utilizes active optical sensing devices (LIDAR and light curtain transmission) to detect, localize, and classify trees. Promising results in our recent work helped system designers select the most reliable sensor for accurate detection of trees in a nursery and to automate labor-intensive tasks, such as weeding, without damaging crops.

In recent years, high-resolution remote-sensing techniques have been utilized in agricultural automation for counting mature trees. An automatic approach for counting olive trees is introduced in [8] with very high spatial remote sensing images. This approach contains two main steps: The olive trees are first isolated from other objects in the image based on a Gaussian process classifier with different morphological and textural features; candidate blobs of olive trees are then considered valid if their sizes are in a range specified by a prior knowledge of the real size of trees. In [9], a method for counting palm trees in Unmanned Aerial Vehicles (UAV) images is presented. To detect palm trees, SIFT keypoints are extracted from the images, and then analyzed by an Extreme Learning Machine (ELM) classifier. The ELM classifier uses prior knowledge trained on palm and non-palm keypoints. The output of this step is a list of palm keypoints that are then merged using an active contour method to produce the shape of each tree. Local binary patterns are used to distinguish palm trees from other objects based on their shapes. A general image processing method for counting any tree in high-resolution satellite images is described in [10]. Steps used in this method include HSI (hue, saturation, intensity) color space transform of the original image, image smoothing, thresholding of the extracted hue image, histogram equalization for HSI channels, candidate region detection, delineation, and tree counting.

This paper presents an uncalibrated camera system for fast and accurate plant counting in an outdoor field and incorporates a single high quality camera, an embedded microcontroller to automate the image capturing process, and a computer vision algorithm. The algorithm includes the steps of orthographic plant projection based on a perspective transform, plant segmentation using excessive green, plant detection by utilizing projection histograms, and plant counting that compensates for overlapping areas between consecutive images (to avoid double-counting). Both the camera and microcontroller were mounted on an ATV (all-terrain vehicle) and the images were analyzed offline.

**Figure 1:**
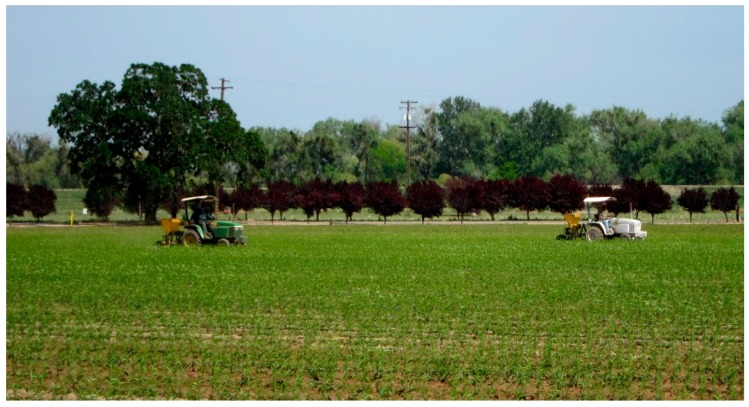
Tractors are performing the fertilization step at the same time as the counting, at Sierra Gold Nurseries, CA, USA.

Compared to previously described systems, the advantages of the proposed system are: It is easy to setup without requiring any camera calibration, it is robust to shadows in the background (e.g., soil, plant residue, and plants in other rows) and, among the target plants, it powerfully copes with noise and foliage occlusion, and is suited for use on a mobile vehicle in the field where row paths are rough and camera vibration common. The ATV platform was used in this paper to simulate the normal orchard tractors that the system was designed to be mount on. The objective was to have a system that can be mounted on a tractor and the counting done as part of the existing field operations. The most compatible existing operation is the fertilization step that is done at the same time as the counting. Figure 1 shows two small tractors performing the fertilization step at the same time as plant counting. This step is compatible because the tractors travel each row and are generating minimal dust. It is also the main argument against a single purpose UAV because by adding a machine vision module onto the front of the tractor shown in Figure 1, we can also count plants in an existing trip of a ground vehicle that is traveling each row.

This paper is organized as follows: In Section 2, we describe the system design and the proposed algorithm. The algorithm is detailed in Section 3 and Section 4 for plant counting for a single image and on an image sequence, respectively. Section 5 presents the experimental results of the system. Finally, this paper draws to a conclusion and discusses future work in Section 6 and Section 7, respectively.

## 2. System Design and Algorithms

**Figure 2:**
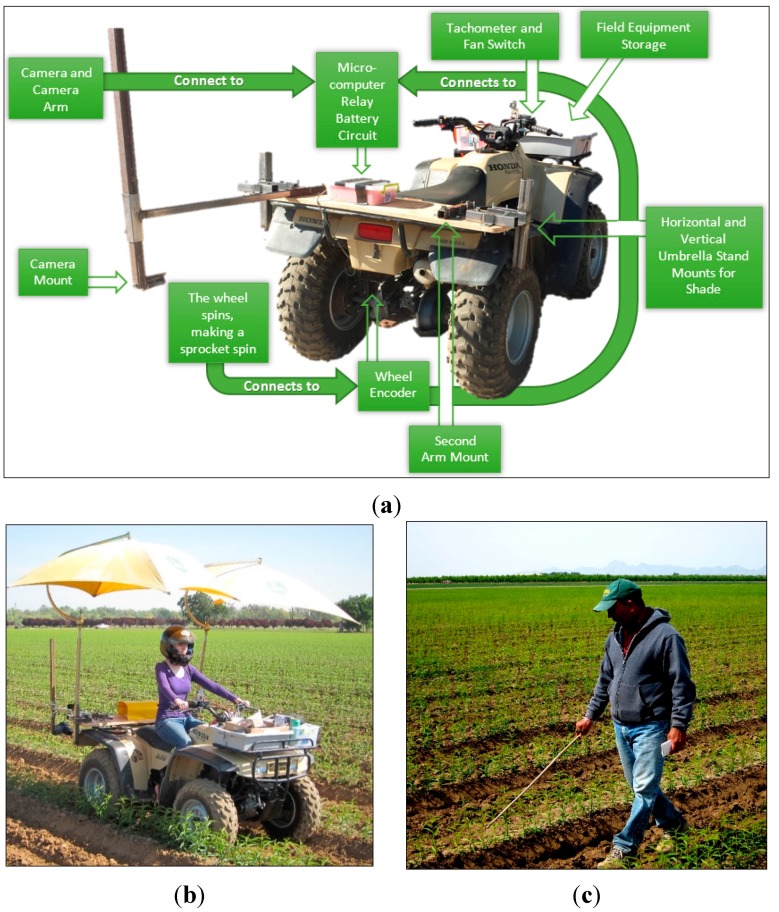
(**a**) The ATV with an arm to mount the camera, the wheel encoder, and the microcontroller to activate the camera via a relay; (**b**) the vehicle in operation capturing pictures of plants; and (**c**) manual counting of plants by a staff person.

In the experimental system an ATV was used to simulate a tractor, as the base platform. The mobile vehicle had an attached arm to hold a camera, and a rotary shaft encoder was mounted on the wheel axle for odometry sensing. Image data are based on 24-bit digital color images taken by an electrically controlled, high-resolution, digital single-lens reflex camera (model EOS Rebel T3, Canon Inc., Tokyo, Japan). The camera was equipped with a zoom lens (model EF-S 18–55 mm 1:3.5–5.6 IS II, Canon Inc., Tokyo, Japan) aimed at the target plants and held fixed on the arm mounted to the mobile vehicle at an orientation of approximately 60° relative to the ground plane. An embedded microcontroller (model Raspberry Pi version 1 model B+, Raspberry Pi Foundation, UK) was used to activate the camera via a solid-state relay. The odometry signal was an input into the microcontroller to control the distance travelled between image acquisition events. A control algorithm was created to allow the microcontroller to trigger image acquisition by the camera at set spatial intervals. Because a difference in image resolution might dramatically affect the processing speed of the whole system from image transfer, plant segmentation, feature detection, and calculation of homography transformation matrix, the resolution 640 × 480 was selected as the best trade-off between speed and quality, so that accurate results can be obtained in an acceptable length of processing time. All camera parameters, including aperture, focal length, shutter speed, white balance, and ISO were manually set to have the best quality images in an outdoor scene. Figure 2 shows all details of the devices on the ATV, including the arm to mount the camera, the microcontroller with a relay and the wheel encoder.

**Figure 3:**
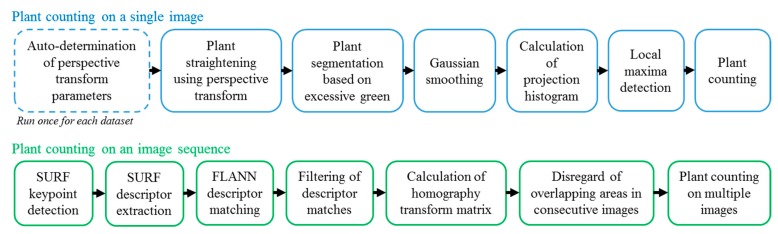
Flowchart representation of the algorithm.

The algorithm contains steps of perspective transform (with automatic determination of parameters) for orthographic plant projection, excessive green color segmentation, Gaussian smoothing, projection histogram, and local maxima detection for plant counting for a single image. To overcome potential problems with double-counting of plants in an image sequence, SURF (Speeded Up Robust Features) keypoint detection, SURF descriptor extraction [11], FLANN (Fast Library for Approximate Nearest Neighbors) descriptor matching [12], filtering of descriptor matches, and calculation of homography transform were utilized with GPU implementations. Figure 3 shows a block diagram of the proposed algorithm.

## 3. Plant Counting in a Single Image

### 3.1. Plant Straightening Using a Perspective Transform

Since the original image is collected using a perspective view, a correction method to straighten the plants (*i.e.*, orthographic projection) for later processing in the steps of projection histogram calculation and local maxima detection is required. Existing studies [13,14] have investigated the affine rectification of the ground plane to make parallel world lines appear parallel in the rectified image. Only one vanishing point is needed in these methods when the camera is at a fixed angle tilting downward toward the ground. This affine rectification method has proven robust to create a virtual orthographic view of the scene. The method can be successfully applied to our system if a vanishing point could be found; however, parallel world landmarks do not exist in the field and thus requires another way of transforming the image. In this section, a perspective transform technique is presented using four predefined points in the source image space and four in the destination image space. The 3 × 3 perspective transform matrix **P** is solved so that:

(1) $$\left[\begin{array}{c} x\prime_{i}\\ y\prime_{i}\\ 1 \end{array}\right]=P\left[\begin{array}{c} x_{i}\\ y_{i}\\ 1 \end{array}\right]=\left[\begin{array}{ccc} p_{11} & p_{12} & p_{13}\\ p_{21} & p_{22} & p_{23}\\ p_{31} & p_{32} & p_{33} \end{array}\right]\left[\begin{array}{c} x_{i}\\ y_{i}\\ 1 \end{array}\right]$$

where *i* = {1, 2, 3, 4}; $\left(x_{i},y_{i}\right)$ and $\left({x^{\prime }}_{i},{y^{\prime }}_{i}\right)$ are the *i*-th points in the source and destination images, respectively. The four points in the source image are defined as

(2a) $$\left(x_{1},y_{1}\right)=(0,0)$$

(2b) $$\left(x_{2},y_{2}\right)=(W-1,0)$$

(2c) $$\left(x_{3},y_{3}\right)=(W-1,H-1)$$

(2d) $$\mathrm{and}(x_{4},y_{4})=(0,H-1)$$

where *W* and *H* are the source image width and height, respectively. The four points in the destination image are calculated based on how the perspective changes with respect to *x* and *y* directions:

(3a) $$\left({x^{\prime }}_{1},{y^{\prime }}_{1}\right)=({\text{δ}}_{x},{\text{δ}}_{y})$$

(3b) $$\left({x^{\prime }}_{2},{y^{\prime }}_{2}\right)=(\text{W}-1-{\text{δ}}_{x},{\text{δ}}_{y})$$

(3c) $$\left({x^{\prime }}_{3},{y^{\prime }}_{3}\right)=(W-1+{\text{δ}}_{x},H-1+{\text{δ}}_{y})$$

(3d) $$\mathrm{and}({x^{\prime }}_{4},{y^{\prime }}_{4})=(-{\text{δ}}_{x},H-1+{\text{δ}}_{x})$$

where δ*_x_* and δ*_y_* express how the perspective is needed to change (see Figure 4). These parameters can be determined automatically using our proposed algorithm that contains all steps from plant straightening to local maxima detection. The algorithm of auto-determination of perspective transform parameters is used only once on the first image of each dataset. The determined δ*_x_* and δ*_y_* are then applied to the remaining images in the set. This algorithm will be described in detail later in this section. Based on the obtained perspective transform matrix **P**, the destination image is transformed by

(4) $$I_{p}\left(x,y\right)=I\left(\frac{p_{11}x+p_{11}y+p_{13}}{p_{31}x+p_{32}y+p_{33}},\frac{p_{21}x+p_{22}y+p_{23}}{p_{31}x+p_{32}y+p_{33}}\right)$$

where *I_p_*(*x*, *y*) and *I*(*x*, *y*) are the destination and source images, respectively. Figure 4 shows a sample image of ten small peach trees before and after performing a perspective transform. In this example, the average inclination angle of plants, with respect to *x*-axis, is significantly corrected from 74.81° to 82.77°.

**Figure 4:**
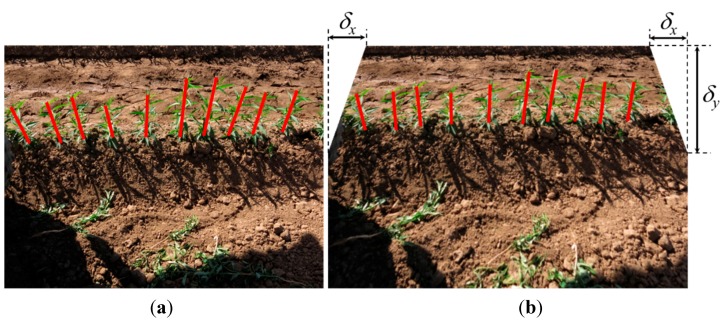
An image before (**a**) and after (**b**) performing a perspective transform for plant straightening. The (**red**) solid lines show the plant inclination. The average inclination angle (with respect to *x*-axis) of the ten plants in the original image (**a**) is 74.81°; and it is 82.77° in the corrected one (**b**).

### 3.2. Plant Segmentation Based on Excessive Green

**Figure 5:**
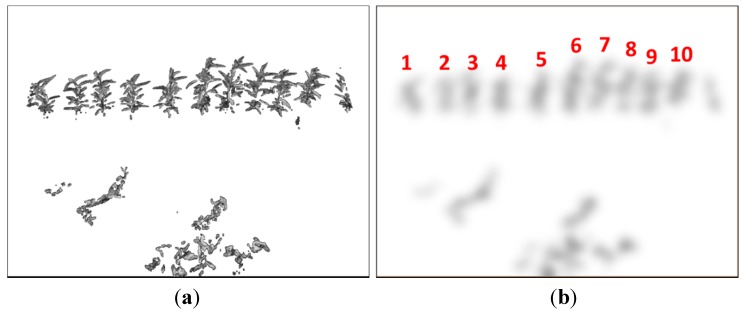
A green-segmented image (**a**) and its Gaussian smoothed image (**b**) where plants are isolated.

Different algorithms for soil, ground, and plant segmentation have been introduced in [15,16,17]. This step herein utilizes a fast and efficient method to segment green objects from the soil or background. The plants and the less-green background in the perspective transformed image were segmented by comparing the excessive green value [17] of each pixel to a certain threshold:

(5) $$G\left(x,y\right)=\{\begin{array}{c} I_{p}\left(x,y\right),\;\;if\;\;\left(2I_{p}^{g}\left(x,y\right)-I_{p}^{r}\left(x,y\right)-I_{p}^{b}\left(x,y\right)\right)>T_{g}\\ 0,\;\;otherwise\;\;\;\;\;\;\;\;\;\;\;\;\;\;\;\;\;\;\;\;\;\;\;\;\;\;\;\;\;\;\;\;\;\;\;\;\;\;\;\;\;\;\;\;\;\; \end{array}$$

where *G*(*x*, *y*) is the green-segmented image; $I_{p}^{g}$, $I_{p}^{r}$, and $I_{p}^{b}$ are the green, red, and blue image channels of *I_p_*(*x*, *y*), respectively; and *T_g_* is a predefined threshold, which was fixed to 40 in this paper. This green color segmentation technique is very fast and effective in our system in segmenting plants from the backgrounds of soil, shadow, or non-green objects. Gaussian smoothing was applied to the thresholded excessive green image to eliminate background noise and reduce the effects of foliage occlusion. In this paper, based on the maximum plant height (approximately 40 cm) and the degree of foliage occlusion from plant to plant, the Gaussian kernel standard deviation size was fixed to 7. Figure 5 presents the green-segmented image of the transformed image in Figure 4 and the corresponding smoothed image.

### 3.3. Projection Histogram and Local Maxima Detection

A vertical projection histogram [14] of the smoothed image is calculated in this section. It is constructed by projecting plant pixels along vertical lines (columns) in a region of interest (ROI). The number of bins in the histogram is equal to the number of columns in the image. In our system, because the camera is fixed on the mobile vehicle, we manually select the ROI to fit all plants in the field row. Let *P^V^*(*G_g_*) be the vertical histogram of the smoothed image *G_g_*, giving

*P^V^*(*G_g_*) = {*h_j_*(*G_g_*): *j* = 0, 1, …, *W* − 1} (6)

Local maxima are detected by finding the histogram bins, where their gray values are greater than their neighbors as

*L*(*i*) = {*h_i_*(*G_g_*): *h_i_*_−*1*_(*G_g_*) < *h_i_*(*G_g_*) and *h_i_*_+*1*_(*G_g_*) < *h_i_*(*G_g_*), *i* = 1, 2, …, *W* − 2} (7)

where *L*(*i*) is the gray value of the local maximum at bin *i*. The number of plants is then determined based on the number of local maxima. It is noted that the detection of the plants at the left and right borders explicitly followed Equation (7) and the definition of ROI, although objects outside the ROI occasionally appear to human eyes, as at the right bottom edges of Figure 5. The (red) numbers in Figure 5 represent the number of detected trees.

### 3.4. Auto-Determination of Perspective Transform Parameters

Notice that this step combines all steps from Section 3.1, Section 3.2 and Section 3.3 and it is done once on the first image of each dataset to have the optimal perspective transform parameters δ*_x_* and δ*_y_* for plant straightening and vertical histogram projection. The parameters δ*_x_* and δ*_y_* are estimated based on a list of detected local maxima as follows:

(8) $$\Delta ({\text{δ}}_{x},{\text{δ}}_{y})=\text{arg}\underset{\begin{array}{c} 0\leq {\text{δ}}_{x}\leq W/4\\ 0\leq {\text{δ}}_{y}\leq H/2 \end{array}}{\max}\overset{n_{L}}{\underset{k=0}{\sum }}L(k)$$

where *n_L_* is the number of local maxima and it is predefined as 10 for the image in Figure 5*.* This step particularly requires proper parameter tuning for the Gaussian filter to smooth the projection histogram and to have less noise in the detection of local maxima.

## 4. Plant Counting in an Image Sequence

Once the number of plants in each image is determined, the accumulated count in all images in a sequence is determined using a special method to avoid double-counting of trees between images. To serve this purpose, the homography transformation between every two successive images is considered. SURF is one of the best algorithms for keypoint detection and descriptor extraction and it is being used successfully in the applications of object recognition [18], image stitching [19], 3D reconstruction [20], and background motion compensation [21,22]. In this paper, SURF was utilized for the calculation of the homography transformation matrix, because it is fast to compute and has good performance when based on integral images and an integer approximation of the determinant of Hessian blob detector. The extracted SURF descriptors are matched between two consecutive images using FLANN, which allows fast and accurate nearest neighbor searches in high dimensional spaces. Furthermore, it can select the optimal matching parameters automatically without any tuning from users. Good matches are thenceforth obtained based on the condition of common distance, *i.e.*, the matches having distances less than a minimum distance threshold will be discarded. In our case, a vehicle is moving along a field row and taking pictures of plants, two consecutive images of the same planar ground surface can be related by homography. The good matches obtained are used as the input to find a homography transformation between each set of successive images, without the need for camera calibration. Once the homography transformation matrix is found, camera translation with respect to vehicle’s horizontal movement can be extracted to estimate the overlap between successive images and avoid double-counting of plants. Defining *I_t_*_−1_(*x*, *y*) = [*x_t_*_−1_, *y_t_*_−1_, 1]*^T^* and *I_t_*(x, y) = [*x_t_*, *y_t_*, 1]*^T^* the image points as time *t* − 1 and *t*, a homography **H** is represented through

(9) $$\left[\begin{array}{c} x_{t}\\ y_{t}\\ 1 \end{array}\right]=H\left[\begin{array}{c} x_{t-1}\\ y_{t-1}\\ 1 \end{array}\right]=\left[\begin{array}{ccc} h_{11} & h_{12} & h_{13}\\ h_{21} & h_{22} & h_{23}\\ h_{31} & h_{32} & h_{33} \end{array}\right]\left[\begin{array}{c} x_{t-1}\\ y_{t-1}\\ 1 \end{array}\right]$$

where *h*_13_ is the coefficient of *x*-translation and that is the parameter we want to utilize for estimating the overlapping between successive images. It is noted that the scaling parameters *h*_11_ and *h*_22_ approach 1 because there are no zoom-in or zoom-out operators from the camera and the vehicle is travelling along the plant row. In our case, an affine homography is considered an appropriate model of image displacement and is a special type of a general homography where *h*_31_ and *h*_32_ are close to 0 and *h*_33_ approaches 1. In this paper, the Hessian threshold for the keypoint detector was fixed to 400, *i.e.*, the features having a Hessian value larger than this threshold are retained. A RANSAC-based method was used to estimate the homography matrix so as to

(10) $$H=\text{arg}\underset{H}{\min}\overset{N}{\underset{i=0}{\sum }}\left[{\left(x_{t}^{i}-\frac{h_{11}x_{t-1}^{i}+h_{12}y_{t-1}^{i}+h_{13}}{h_{31}x_{t-1}^{i}+h_{32}y_{t-1}^{i}+h_{33}}\right)}^{2}+{\left(y_{t}^{i}-\frac{h_{21}x_{t-1}^{i}+h_{22}y_{t-1}^{i}+h_{23}}{h_{31}x_{t-1}^{i}+h_{32}y_{t-1}^{i}+h_{33}}\right)}^{2}\right]$$

where *N* is the total number of keypoints and $\left(x_{t}^{i},y_{t}^{i}\right)$ is the image point *i* at time *t*. Local maxima positions of plants are then compared to the *x*-translation parameter *h*_13_ to determine whether they are double-counted. Due to the effect of camera distortion (i.e., the plants at the image border are less straight than those closer to image center), this determination step requires a “buffer” for the plants at the image border to reduce the amount of counting errors. When *h*_13_ > 0, the comparison (*i_l_* − *b* > *h*_13_) implies that the plant at the local maxima position *i_l_* (obtained from the Equation (7)) is considered double-counted if its difference from the buffer *b* is greater than *h*_13_. When *h*_13_ ≤ 0, the comparison (*i_l_* + *b* < *W* + *h*_13_) is to check if the plant at *i_l_* is double-counted, where *W* is image width. The buffer *b* can be estimated automatically based on half of the average of distances between two neighbor local maxima.

## 5. Experimental Results

A computer (CPU model Core i7 at 3.4 GHz, Intel Co., Santa Clara, CA, USA, with 12-GB DDR3 random-access-memory) was used for all processing steps, except that an 1152 core GPU (model GeForce GTX 760, NVidia Co., Santa Clara, CA, USA) graphics card was utilized for implementing the GPU-based SURF descriptor extraction and FLANN matching algorithms. Experiments were conducted on 941 images containing 9915 juvenile *Prunus* *persica* L. “Nemaguard” peach trees (approximately 10.54 plants per image). Accounting for image overlap, the 941 images contained 2178 distinct peach trees when double-counting was eliminated. The images of the juvenile peach trees were collected from seven rows, with a 10 cm in-row plant distance between two neighboring plants, in an outdoor tree nursery (Sierra Gold Nurseries, Yuba City, CA, USA). Table 1 shows information for the datasets used in the experimental results, where Sets 1, 2, and 3 (namely, Group 1) were taken on the same day and Sets 4 to Set 7 (namely, Group 2) were acquired on another day. The maximum plant height was approximately 30 cm, and 40 cm in Groups 1 and 2, respectively. The overlap between two consecutive images was increased from approximately 60% in Group 1 to approximately 90% in Group 2. These plant images were selected as examples of plants covered by shadow (from human, other plants, or random objects), green and other objects in the background, small plants, and different plant sizes.

**Figure 6:**
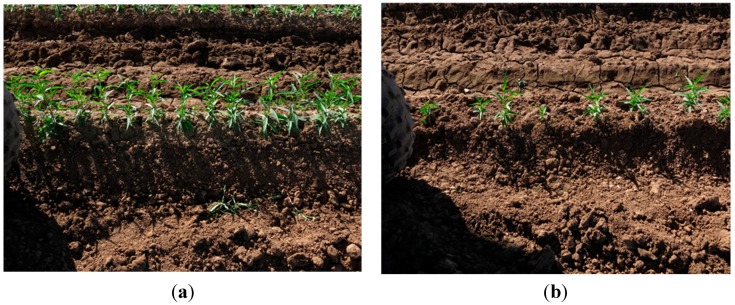
Sample images of green objects in the background (**a**) where there is plant residue on the soil and plants in the next row; and small plants (**b**).

Figure 6 presents sample images showing examples of the green plant residue between the rows of plants, a second row of trees at the back (Figure 6a) and eight small plants (Figure 6b). In this study, the ground truth number of plants and overlap of images were measured manually. The number of small plants was defined based on a maximum plant height of 15 cm. It is noted that there were no plants imaged in shadow from Sets 3 to 7, and no additional objects on the background in Set 3. In the final design of our system on a tractor, a metal tunnel will be used to eliminate the current issues of shadows and green objects (*i.e.*, the adjacent row) in the background. The purpose of presenting results of these critical issues here was to experimentally show the robustness of the proposed algorithm. In the experiments, all parameters were fixed (*i.e.*, not optimized image by image) in order to have consistent results between different images and datasets.

**Table 1:** List of datasets and their detail information for experiments.

		Set 1	Set 2	Set 3	Set 4	Set 5	Set 6	Set 7	Total
**# images**	All	121	127	93	150	150	150	150	941
	Containing shadows	18	3	0	0	0	0	0	21
	Containing green material on the background	6	10	46	150	150	150	150	662
	Containing other objects on the background	3	5	0	13	13	6	9	49
	Containing small plants	18	21	12	6	5	11	34	107
**# plants**	In individual images	1469	1481	1154	1398	1402	1505	1506	9915
	In the image sequence	609	505	525	160	136	94	149	2178
	With shadows in individual images	46	7	0	0	0	0	0	53
	Of small size in individual images	26	42	13	6	5	15	53	160
**Average image overlap**		59.2%	64.7%	53.8%	89.4%	91.1%	93.8%	90.4%	

**Figure 7:**
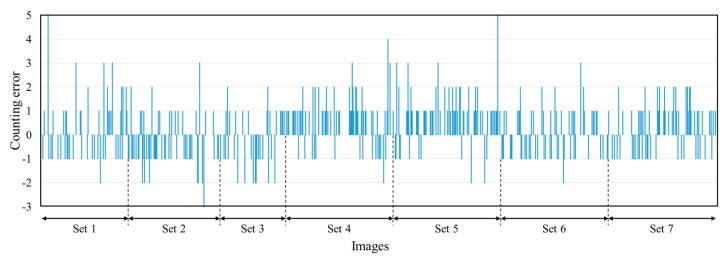
Tree counting errors in 941 images.

Table 2 shows the average plant counting accuracies in both plant counting within a single image and for an image sequence, where double-counting was avoided. Within a single image, on average 0.51 counting mistakes out of 10.54 plants per image (*i.e.*, an average accuracy of 95.2%) were observed. Accuracies obtained when excluding the challenging cases 95.4% (shadows), 95.4% (additional green objects), 95.3% (other objects), and 95.3% (small plants) were approximately equivalent to the overall performance, demonstrating the robustness of the method. Notice that the underlined numbers in Table 2 for the cases of “shadow” and “green objects” were taken from those without the exclusion. In an image sequence, when double-counting was minimized, an accuracy of 98% in all images was achieved. This accuracy is better than the single image performance because, on average, errors in a sequence of overlapped images compensate for one another. The plant counting error in every image is shown in Figure 7. The maximum estimation error was five plants and was mostly associated with high levels of foliage occlusion. By estimating the homography transformation between consecutive images, the proposed system also produced knowledge on the amount of image overlap. Comparing the actual overlap to that estimated by homography, we obtained an average error of only 2.54%. The average processing time for all steps in the software algorithm was approximately 300 ms per image pair.

**Table 2:** Average counting accuracy and the estimated image overlap using a homography transform.

		Set 1	Set 2	Set 3	Set 4	Set 5	Set 6	Set 7	Avg.*
**Within single images**	w.r.t. individual images	95.8%	95.5%	96.4%	93.8%	93.6%	95.8%	95.3%	95.2%
	Avg. count errors per image	0.51	0.52	0.44	0.57	0.60	0.42	0.47	0.51
	Std. Dev. of count errors per image	0.82	0.67	0.67	0.74	0.83	0.59	0.63	0.72
	Excluding the case of shadows	96.2%	95.8%	96.4%	93.8%	93.6%	95.8%	95.3%	95.4%
	Excluding the case of green objects on the background	95.7%	95.5%	96.7%	-	-	-	-	95.4%
	Excluding the case of other objects on the background	95.6%	95.6%	96.4%	93.9%	93.7%	96.1%	95.8%	95.3%
	Excluding the case of small plants	95.8%	95.4%	96.5%	94.0%	93.9%	96.2%	95.4%	95.3%
**For an image sequence**		99.2%	98.2%	99.2%	96.3%	95.6%	99%	98.6%	98%
**Estimated image overlap**		57.1%	62.1%	58.7%	87.6%	88.6%	91.7%	88.6%	

*: The final average values were calculated across all datasets.

Comparisons in terms of counting accuracy and system characteristics between our method and the other two methods in [4,6] (where corn plants were used) are shown in Table 3. The results are presented with respect to counting in individual images, and for an image sequence using our peach tree data. In the method [4], the block matching based image sequencing algorithm was not applicable to our data, which were captured from a camera held at an angle of 60°. Due to a large number of incorrect matches yielded by the block matching based algorithm, our image sequencing algorithm (without the perspective transform) was substituted for the block matching in [4] to allow comparison of subsequent steps. The accuracies yielded by [4] were significantly lower than ours. The iterative rules based on the number of pixels and positions were sensitive to plant size and plant center locations. Additionally, these rules required parameter tuning for refining plant and background regions. Similarly, in the method from [6], when the top view was used, it led to plant counting errors when the direct image sequencing was utilized without the perspective transform. Using skeletonization for plant center detection, the method in [6] yielded a high error rate when there was foliage occlusion. For individual images, our system yielded an average accuracy of 95.2% (0.51 ± 0.72 count errors per image) compared to 86.9% (1.38 ± 1.34) for the method of [4] and 71.4% (2.05 ± 1.96) for the method in [6]. In the case of the image sequence, our total accuracy (98%) was substantially better than those of the other two methods (77.8% and 71.9%). It is worth mentioning that our method was able to work well with less than 60% image overlapping compared to 85% of [4].

**Table 3:** Accuracy and system characteristics comparison of the proposed method to [4,6].

		The Method of [4]	The Method of [6]	The Proposed Method
**Accuracy comparison (using peach tree seedling data)**				
**Within individual images**	w.r.t. individual images	86.9%	71.4%	95.2%
	Avg. count errors per image	1.38	2.05	0.51
	Std. Dev. of count errors per image	1.34	1.96	0.72
**In an image sequence**		77.8%	71.9%	98%
**System characteristics**				
**Plant size (growth stage)**		V3 to V4 growth stages *	Early to V3 growth stages *	Early growth stage to 40 cm height
**Camera view**		Top view	Top view	Perspective view at an angle of 60°
**Image overlap**		85%	n/a	54% to 91%
**Method**	Image sequencing	Block matching (substituted by our image sequencing method without perspective transform)	SIFT feature matching, homography transform	SURF descriptor extraction, RANSAC feature matching, homography transform
	Plant segmentation	Bayesian classification on color spaces	Bayesian classification on color spaces	Excessive green
	Plant counting	Iterative rules based on the number of pixels and positions	Skeletonization for plant center detection	Perspective transform, Gaussian smoothing, projection histogram

*: The V3 growth stage in corn implies three leaves with visible leaf collars.

## 6. Conclusions

A digital imaging system for tree crop enumeration was successfully developed and tested. The system uses an embedded microcontroller mounted on an ATV (to simulate a tractor that will be used in actual practice the field) to receive the odometry signal and trigger the image acquisition. In this paper, the ability to automatically count a large number of small peach trees, with no human effort compared to manual counting, was demonstrated. The estimated count allows confirmation of the germination rate and final plant stand prior to budding in tree seedlings grown in outdoor nurseries. For the juvenile trees to a 40 cm height and 10 cm within-row plant spacing, the method successfully counted plants with an average accuracy of 95.2% in individual images and 98% for counting the whole plant population in a long image sequence. The method also provides robust performance for the cases of plants in shadow, green and other colored objects on the image background, small plants, and foliage occlusion.

## 7. Future Work

**Figure 8:**
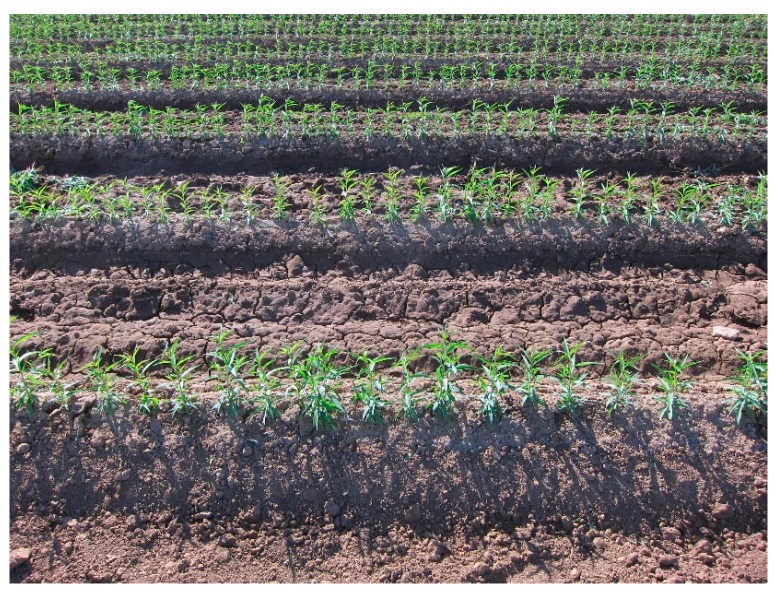
Sample image of rows for the purpose of counting multiple rows simultaneously.

Future work should include (1) automating the system to perform real-time analysis and plant counting in the field; (2) improving and optimizing the algorithms to support larger seedlings; (3) counting multiple rows (Figure 8) simultaneously for a redundant check on the count or allowing a count with fewer passes through the field; (4) supporting measurements of stem width and plant height; and (5) plant phenotyping for tree crops in seedling nurseries and for mapping the location of each plant for individual plant care.
